# Effects of extracorporeal membrane oxygenation and/or renal replacement therapy on piperacillin/tazobactam levels in critically ill patients

**DOI:** 10.3389/fphar.2026.1725502

**Published:** 2026-05-07

**Authors:** T. Riese, S. Lauter, G. Surat, M. Kurlbaum, D. Roeder, P. Meybohm, K. Hoppe

**Affiliations:** 1 Department of Anaesthesiology, Intensive Care, Emergency and Pain Medicine, University Hospital Würzburg, Würzburg, Germany; 2 Unit of Infection Control and Antimicrobial Stewardship, University Hospital Würzburg, Würzburg, Germany; 3 Department of Internal Medicine I, Division of Endocrinology/Diabetology, University Hospital Würzburg, Würzburg, Germany; 4 Central Laboratory, Core Unit Clinical Mass Spectrometry, University Hospital Würzburg, Würzburg, Germany

**Keywords:** continuous renal replacement therapy, CRRT, ECMO, extracorporeal therapies, personalized antimicrobial therapy, PIP/TAZ, therapeutic drug monitoring

## Abstract

**Background:**

Although organ replacement therapy serves as a bridge to the recovery of vital organ function, it may lead to significant physiological and pharmacokinetic changes. To identify factors that may affect patient outcomes due to inadequate antimicrobial therapy, we aimed to evaluate the effectiveness of primary dosage determination of piperacillin (PIP) in the intensive care unit (ICU) setting, particularly in patients undergoing renal or lung replacement therapy.

**Methods:**

Between January 2020 and December 2021, we retrospectively analyzed all ICU patients with a confirmed SARS-CoV-2 infection who received piperacillin/tazobactam (PIP/TAZ) continuous infusion due to suspected secondary infection. The analysis included a comparison of serum PIP levels, demographic data, and laboratory findings among different patient groups.

**Results:**

From 108 included patients, therapeutic drug monitoring (TDM) was performed at the initiation of antibiotic treatment in 91 (84.3%) cases. Of these patients, 15 received extracorporeal membrane oxygenation (ECMO), 12 underwent continuous renal replacement therapy (CRRT), and 43 received both procedures during their ICU stay. The median age was 58 years. The in-hospital mortality rate was significantly higher in patients receiving ECMO therapy (73.3% vs. 23.8%) and in those receiving both ECMO and CRRT (72.1% vs. 23.8%) compared to patients without organ replacement procedures. Dosing calculation adjusted to renal function was correctly performed according to the established standard operating procedure in 96.7% of cases. The PIP level after primary dosage, prior to further adjustments, was within the standard operating procedure-defined concentration range of 32–96 mg/L, encompassing the empirical target range and the higher exposure range relevant in specific clinical scenarios, in 59.0% [median target concentration: 44.0 mg/L] of patients without organ support, 58.3% of those on ECMO [median target concentration: 66.8 mg/L], 80.0% of those undergoing CRRT [median target concentration: 56.0 mg/L], and in 27.8% of the patients receiving both ECMO and CRRT [median target concentration: 116.0 mg/L]. In total, 17.6% of all patients did not reach the minimum PIP target level of 32 mg/L and 27.5% were above the maximum PIP target level of 96 mg/L. Binary regression analysis did not identify significant correlation between plasma bilirubin, plasma albumin, the capillary leak index (CLI) or anthropometric variables and achieving the target plasma concentration of PIP.

**Conclusion:**

In total, 45% of first-dose PIP concentrations in the analyzed critically ill patients were outside the recommended concentration corridor. While lowest concentrations of PIP levels were detected in critically ill patients without organ support therapy, levels above the therapeutic range were detected in patients treated with ECMO support. However, based on the data of this study, a predictor to prevent primary drug levels outside the therapeutic range was not identified. These findings underscore the critical role of TDM in daily clinical routine.

## Introduction

In accordance with the Third International Consensus Definition, sepsis is defined as a life-threatening organ dysfunction resulting from a dysregulated host responses to infection (Singer et al., 2016). While some impairments, such as those affecting coagulation or the cardiovascular system, are primarily managed with medical treatment, others–such as renal or respiratory failure–require mechanical support. The prevalence of acute kidney injury (AKI) is reported to exceed 50% of patients on ICU with sepsis being the leading reported aetiology. A multi-centre study described renal replacement therapy in up to ten percent of all identified ICU patients (Hoste et al., 2015). An analysis of 459 ICUs reported the prevalence of ARDS in more than ten percent of all admissions (Bellani et al., 2016; Meyer et al., 2021), with some cases requiring rescue therapies like extracorporeal membrane oxygenation. Although, organ replacement therapy as an important achievement of modern intensive care medicine contributes to bridge vital organs, it may lead to several physiological and pharmacokinetic changes due to altered elimination, dilution and secondary influences of hemodynamics. It is well documented that inadequate antibiotic therapy is associated with an increased risk of mortality with each passing hour of delay (Cecconi et al., 2018; Kumar et al., 2006). Studies have shown that a significant portion of dosing intervals did not meet the predetermined minimum therapeutic target concentrations of frequently used broad-spectrum antibiotics like PIP during continuous renal replacement therapy (Roberts et al., 2012; Jamal et al., 2014) and among ECMO treatment (Kühn et al., 2020). The paucity of data concerning patients undergoing intensive care for SARS-CoV-2 infection and treated with antimicrobials due to a suspected secondary infection represents a significant limitation in the current literature. Therefore, we retrospectively evaluated these patients on organ replacement therapy treated with PIP/TAZ on our intensive care unit between 2020 and 2021.

## Methods

### Study design and location

This report presents the findings of a descriptive retrospective single-center study. The study included patients with confirmed SARS-CoV-2 infection who were treated with PIP/TAZ due to suspected sepsis in accordance with a previously published protocol (Nikolas et al., 2021). All patients included in the study were treated on the intensive care unit of the University Hospital Würzburg, Germany, between January 2020 and December 2021. The data were captured and recorded in accordance with standard protocols within the hospital information system and included patient data such as demographics, clinical characteristics, laboratory results, procedures, and follow-ups. The study was carried out in accordance with Declaration of Helsinki and Declaration of Taipei. Data analysis and reporting was performed anonymously. As retrospective report the approval was waived by the Institutional Review Board of the Medical Faculty, University Würzburg, Germany (No.: 20210929 03).

### Inclusion and exclusion criteria

The study population included adult patients with a confirmed SARS-CoV-2 infection who were admitted to the intensive care unit and received at least one dose (4.5 g) of PIP/TAZ due to a suspected secondary bacterial infection.

### Data and blood sample collection

In order to facilitate the early identification of sepsis in suspicious patients, all patients were subjected to clinical observation on a 24-h basis by attending physicians and nurses. Furthermore, twice daily, experienced intensive care specialists visited the patient. The decision to commence antimicrobial treatment was based on a combination of clinical and laboratory findings, in accordance with the standardized regime previously outlined. On a biweekly basis, the antimicrobial stewardship team provided support to clinicians engaged in the continuous reevaluation and adaptation of their therapeutic antibiotic regimens.

The attending physician obtained anamnestic data on the day of admission and updated it as subsequent findings occurred. Clinical parameters were largely automatically recorded in the hospital information system and manually checked for plausibility. Established scores were used daily to assess the patient’s condition and evaluate the healing process. Blood samples were taken after admission and daily at 4:00 a.m. during the entire stay until discharge or death, and analyzed no later than 2 hours after collection.

### Renal impairment and CRRT

In patients not receiving CRRT, renal function was considered stable enough to allow estimation of GFR using the CKD-EPI equation. In patients with dynamic renal dysfunction or undergoing CRRT, urine output data were not available; residual kidney function was therefore assessed primarily based on serum creatinine measurements, while acknowledging the limited interpretability of creatinine-based eGFR during ongoing CRRT. Because urine output data were not consistently available, formal AKI classification according to KDIGO criteria was not feasible in this retrospective cohort. CRRT was performed as CVVHD with regional citrate anticoagulation, using standard settings of 100 mL/min blood flow and 2000 mL/h dialysate flow unless otherwise specified.

#### Dosing standard and therapeutic drug monitoring

The dosing standard applied in this study followed the institutional standard operating procedure (SOP) for PIP/TAZ administration via continuous infusion. All patients received a loading dose of 4.5 g over 30 min, followed immediately by continuous infusion; no intermittent maintenance infusion regimen was used. According to a previously published decision pathway (Nikolas et al., 2021), the maintenance dose was determined based on renal function. Patients with an eGFR ≤20 mL/min/1.73 m^2^ received 9 g PIP/TAZ per 24 h, whereas those with eGFR >20 mL/min/1.73 m^2^ or undergoing renal replacement therapy received 13.5 g per 24 h. eGFR was calculated using the CKD-EPI equation.

The dosing regimen and the performance of TDM were assessed to evaluate adherence to the SOP-defined indications.

Sample stability was verified during method validation, showing minimum stability of 5 h at room temperature and 8 h at 2 °C–8 °C. The in-house measurement of antibiotics like PIP helps to avoid sample instability and is an essential component of valid and reliable results on the same day. As continuous infusion was used, steady-state conditions were reached within several hours after the loading dose. The first PIP level measurement was performed at least 6 h after the loading dose to approximate steady state exposure; therefore, detailed sampling times were not reported.

For this study, samples were obtained exclusively from blood. PIP levels were determined by isotope dilution HPLC (high-performance liquid chromatography) tandem mass spectrometry (HPLC-MS/MS), following a previously published process (Nikolas et al., 2021). PIP exhibits time-dependent antibacterial activity, and the most relevant PK/PD index is the fraction of time during which the unbound concentration remains above the minimum inhibitory concentration (fT > MIC). In critically ill patients, PK/PD targets such as 100% fT > MIC or higher targets have been discussed. However, only total PIP concentrations were measured in this retrospective analysis. In most cases, the guidelines specify target ranges for PIP but not for tazobactam (Deu et al., 2020). Therefore, our assessment was based on a predefined pragmatic total-concentration target rather than on direct evaluation of free-drug PK/PD target attainment.

### Subgroup definition

1. Organ support–based groups (Groups 1–4)

For analyses of overall clinical characteristics, patients were categorized into four groups according to organ support received at any time during the ICU stay: Group 1 (no ECMO/CRRT), Group 2 (ECMO only), Group 3 (CRRT only), and Group 4 (ECMO + CRRT).

2. Antibiotic therapy–based groups (Groups A–D)

For analyses focusing on antibiotic therapy and PIP/TAZ target attainment, patients were reclassified into four groups according to the organ support present at the start of antibiotic therapy: Group A (no ECMO/CRRT), Group B (ECMO only), Group C (CRRT only), and Group D (ECMO + CRRT). In contrast to Groups 1–4, which reflect organ support at any time during the ICU stay, Groups A–D reflect organ support specifically at PIP/TAZ initiation.

### Statistical analysis

All data were extracted from the hospital information system into a database and anonymized. Calculations and statistical analysis were performed using IBM SPSS Statistics version 29.0.0.0 (IBM, Armonk, NY, United States). Data were presented as frequency distributions and percentages. All continuous data were presented as median ± interquartile range (IQR). Differences between groups were assessed for statistical significance using the Mann-Whitney U-test respectively the Kruskal-Wallis-Test in case of more than two groups and were considered as significant as p ≤ 0.05. Normal distribution was tested using the Kolmogorov-Smirnov-Test. The coefficient of correlation (ρ) was calculated using the Spearman Correlation analysis. Logistic regression analysis was applied to evaluate the association between clinical patient data and PIP levels. Differences in categorical variables between the four ECMO/CRRT subgroups were analysed using Pearson’s chi-square test. Effect size was calculated using Cramer’s V, and the achieved statistical power for the chi-square test was determined based on the observed effect size. For the multivariable logistic regression analysis, the following variables were included: Sequential Organ Failure Assessment (SOFA) score, Simplified Acute Physiology Score II (SAPS II) score, bilirubin, albumin, norepinephrine dose, CLI, age and BMI. Multicollinearity between SOFA and SAPS II was excluded (Pearson r = 0.507; Condition Index 9.53). Both variables were therefore retained in the multivariable model. eGFR was not included due to the lack of a validated method to estimate GFR in patients receiving CRRT or in AKI.

## Results

### Study population

Between January 2020 and December 2021, we identified 113 patients with suspected secondary bacterial infection who received PIP/TAZ. This represents 51% of all patients (n = 221) treated for Sars-CoV-2 during this period. After excluding five patients due to short-term stays, 108 patients were further analysed. TDM was performed at the initiation of antibiotic treatment in 91 (84.3%) patients, who were finally included in this study. The median age of the included population was 58 years (IQR 48–64 years), the median height was 175 cm (168–180 cm) and the median weight was 90 kg (80–107 kg) resulting in a median body mass index (BMI) of 29.3 kg/m^2^ (26.3–34.5 kg/m^2^).

A total of four subgroups were analyzed based on organ support requirements:

Group 1 (n = 21; 23%) received neither ECMO nor CRRT;
Group 2 (n = 15; 17%) received ECMO only;
Group 3 (n = 12; 13%) received CRRT only;
Group 4 (n = 43; 47%) received both ECMO and CRRT.

Using the organ support–based classification (Groups 1–4), no significant differences were observed regarding age and BMI (Table 1).

**TABLE 1:** Demographic characteristics according to organ support–based groups (Groups 1–4), defined by ECMO/CRRT use at any time during the ICU stay.

At any time	Group 1ECMO−/CRRT−(n = 21; 23%)	Group 2ECMO+/CRRT−(n = 15; 17%)	Group 3ECMO−/CRRT+(n = 12; 13%)	Group 4ECMO+/CRRT+(n = 43; 47%)	p
Age [years]	60 (53–64)	57 (38–63)	59 (37–76)	60 (49–65)	0.602
BMI [kg/m^2^]	27.7 (24.8–31.1)	29.2 (26.1–43.4)	29.3 (25.4–32.4)	29.3 (27.5–35.2)	0.462
Male sex, n (%)	14 (66.7)	10 (66.7)	10 (83.3)	27 (62.8)	0.780

Data are presented as median (IQR). The last column contains the p-value comparing distribution over all groups (Kruskal–Wallis test for continuous variables; chi-square test for sex).

### Outcomes

The overall mortality rate was 54.9%. Within the organ support–based classification (Groups 1–4), overall ICU mortality differed significantly between groups. The lowest mortality rate, 23.8%, was observed in Group 1 (n = 21). Among patients who underwent CRRT, Group 3 (n = 12) had a mortality rate of 25.5%. The highest mortality rates were observed in Group 2 (n = 15) and Group 4 (n = 43), which included patients requiring ECMO therapy, at 73.3% and 72.1%, respectively. This was significantly higher than in Group 1. Regarding the overall duration of the ICU treatment, which was a median of 15 days (IQR: 10-25) across all groups, a notable discrepancy was observed between Group 4 and Group 1 (Table 2).

**TABLE 2:** Outcome according to organ support–based groups (Groups 1–4), defined by ECMO/CRRT use at any time during the ICU stay.

At any time	Group 1ECMO−/CRRT−(n = 21; 23%)	Group 2ECMO+/CRRT−(n = 15; 17%)	Group 3ECMO−/CRRT+(n = 12; 13%)	Group 4ECMO+/CRRT+(n = 43; 47%)	p
Mortality	23.8% (5)	73.3% (11), **p = 0.004**	25.0% (3), p = 0.956	72.1% (31), **p < 0.001**	p < 0.001
ICU time [d]	12 (9–20)	12 (8-19), p = 1.000	16 (8-25), p = 0.726	17 (10-27), p = 0.119	p = 0.333

Mortality of all included patients (n = 91) was 54.9%. The term “ICU time” refers to the duration (median (IQR)) of an individual’s hospitalization in the intensive care unit (ICU) and was 15 (10-25) over all groups.

P-values represent the differences compared to Group 1. The last column contains the p-value comparing distribution over all groups (Kruskal–Wallis test).

### Implementation of therapeutic drug monitoring

**FIGURE 1:**
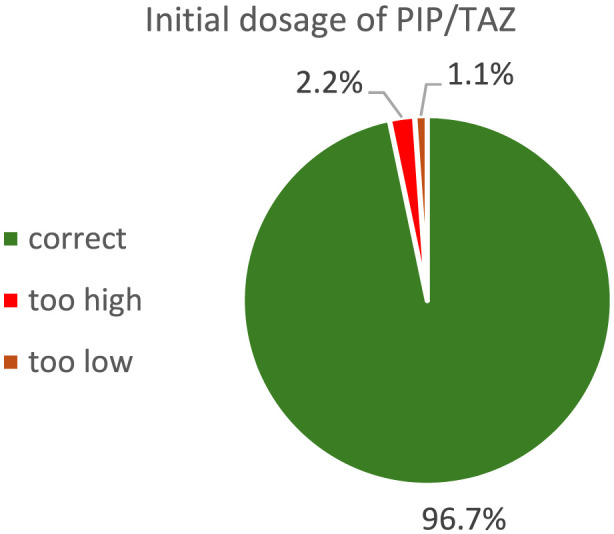
Dosage choice of PIP/TAZ depending on renal impairment and CRRT for all cases following the SOP-defined dosing algorithm.

**FIGURE 2:**
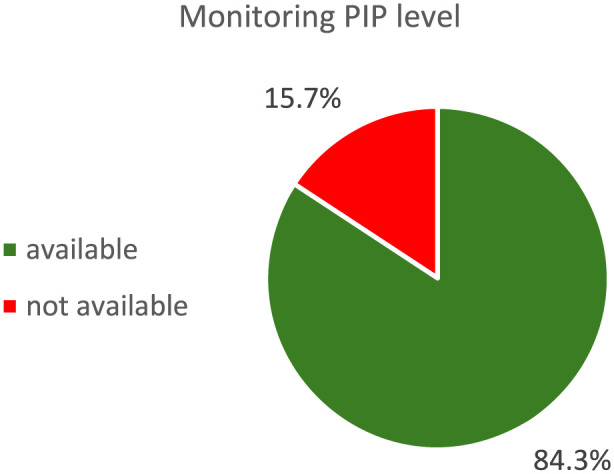
Percentage of patients with documented PIP level after primary application.

Compliance with the SOP-defined dosing algorithm was very high: in 88 of 91 cases (96.7%) the administered dose matched the recommended regimen; in two cases (2.2%) it was higher and in one case (1.1%) lower than recommended (Figure 1). The median administered maintenance dose was 13.5 g per 24 h. PIP level measurements were correctly performed in 91 of the 108 cases with an indication for TDM according to the SOP; in 15.7% of cases, no PIP level was documented (Figure 2).

### Piperacillin levels

For the subgroup analysis focussing on antibiotic therapy, all patients were newly allocated into four different groups (A-D) based on the use of ECMO or CRRT. In contrast to the previous classification, which considered the use of organ replacement procedures at any time point during the entire intensive care unit stay, the following classification accounts for organ replacement therapy that was actually in place at the beginning of antibiotic therapy (Table 3).

**TABLE 3:** Clinical and laboratory characteristics at the start of antibiotic therapy according to antibiotic therapy–based groups (Groups A–D), defined by ECMO/CRRT status at treatment initiation.

At start of antibiotic therapy	Group A ECMO –/CRRT – (n = 39; 43%)	Group B ECMO +/CRRT – (n = 24; 26%)	Group C ECMO –/CRRT + (n = 10; 11%)	Group D ECMO +/CRRT + (n = 18; 20%)	p
Albumin [g/dL]	2.7 (2.4–2.9)	2.6 (2.2–3.0), p = 0.336	2.4 (2.1–2.7), p = 0.062	2.8 (2.5–3.1), p = 0.395	0.128
CLI	53.1 (9.7–91.4)	92.8 (46.2–126.0), **p = 0.043**	100.8 (63.7–119.2), **p = 0.020**	76.0 (64.5–103.0), **p = 0.046**	**0.031**
eGFR [mL/min/1.73m^2^]	76 (65–107)	93 (55-103), p = 0.456	**n.d.** ^a^	**n.d.** ^a^	**n.d.** ^a^
Bilirubin [mg/dL]	0.60 (0.43–0.90)	0.80 (0.53–2.1), p = 0.075	0.75 (0.58–1.52), p = 0.251	1.30 (1.10–2.10), **p < 0.001**	**0.002**
SOFA score	12 (10–16)	14 (12-16), p = 0.092	13 (10-17), p = 0.478	16 (14-17), **p = 0.001**	**0.008**
SAPS II score	59 (48–68)	59 (46-76), p = 0.825	62 (45-77), p = 0.711	69 (62-80), **p = 0.009**	0.097

The measured values for the albumin and bilirubin levels are shown, as well as the calculated eGFR and the CLI, defined as C-reactive protein (CRP) over albumin ratio, multiplied by 100. SOFA score and SAPS II score were calculated at the start of antibiotic therapy. All values are presented as median and interquartile range. P-values represent the differences compared to Group A. The last column contains the p-value comparing distribution over all groups (Kruskal–Wallis test).

^a^ n.d., not determined. Due to CRRT, creatinine levels fluctuate, subsequently the assumption of stable serum creatinine levels, which is necessary to calculate eGFR, is invalid.

The comparison between groups was intended to describe early piperacillin exposure under clinically relevant organ support constellations present at the start of antibiotic therapy, rather than to establish formal pharmacokinetic equivalence between groups.

In CRRT-treated patients (Groups C and D), the prescribed CVVHD settings (dialysate flow 2000 mL/h) corresponded to a median prescribed effluent dose of 21 (IQR 18–25) mL/kg/h based on admission body weight. In patients receiving ECMO (Groups B and D), extracorporeal support was exclusively provided as veno-venous ECMO (vv-ECMO).

The determined median PIP level for all patients was 65.0 (36.2–105.0) mg/L.

- Group A (without ECMO or CRRT support) had a median level of 44.0 (29.9–72.8) mg/L.

- Group B (under ECMO therapy) showed a median level of 66.8 (42.8–120.0) mg/L.

- Group C (under CRRT) had a median PIP concentration of 56.0 (41.2–81.6) mg/L.

- Group D (under both ECMO and CRRT support at start of antibiotic treatment) had a median PIP, level of 116.0 (79.6–137.5) mg/L.

The PIP levels in Group B (p = 0.012) and Group D (p < 0.001) differed significantly from those in Group A (Table 3).

**FIGURE 3:**
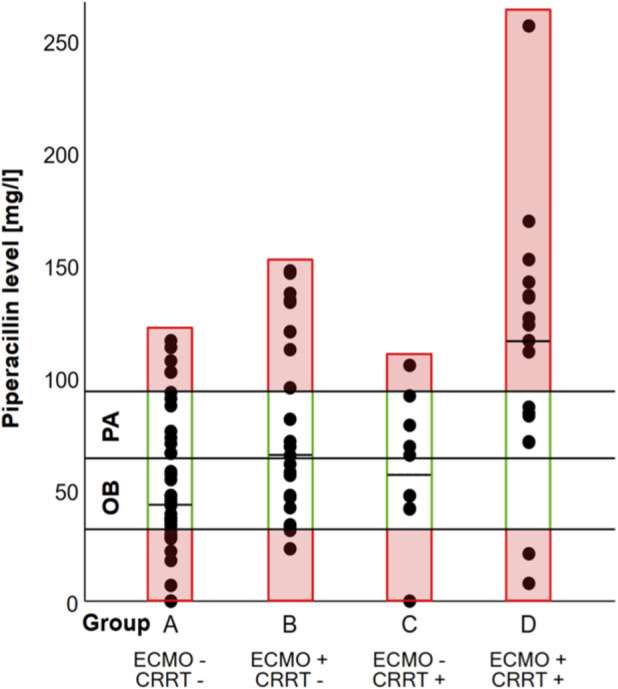
The distribution of PIP concentrations in patients undergoing organ replacement therapy is shown. Black horizontal lines indicate concentration thresholds at 32, 64, and 96 mg/L. The range labelled “OB” represents the empirical target range for other bacteria (32–64 mg/L) according to the institutional SOP, whereas the range labelled “PA” represents the higher target range for *Pseudomonas aeruginosa* (64–96 mg/L). Coloured rectangles indicate whether the group-specific median concentration was within or outside the respective SOP-defined concentration ranges. The dashed line denotes the median concentration.

According to the institutional SOP, the empirical target range for PIP serum concentration was 32–64 mg/L. In specific clinical scenarios, particularly in suspected or proven *Pseudomonas aeruginosa* infection, a higher target range of 64–96 mg/L was considered relevant (Richter et al., 2019; Chiriac et al., 2021). Using this definition, the target serum concentration was achieved in 38.5% of patients in Group A, 37.5% in Group B, and 40.0% in Group C. No patients in Group D achieved the target serum concentration. In addition, because the SOP considers higher exposures relevant in specific clinical scenarios, particularly in suspected or proven *P. aeruginosa* infection, we also descriptively assessed the broader SOP-defined concentration range of 32–96 mg/L. Using this broader range, target attainment was observed in 59.0%, 58.3%, 80.0%, and 27.8% of patients in Groups A–D, respectively (Figure 3).

Using the empirical target range of 32–64 mg/L, the distribution of target attainment across the four groups (A–D) differed significantly (χ^2^(3) = 9.99, p = 0.019). The effect size according to Cramer’s V was 0.33, indicating a medium association between group allocation and target attainment.

A total of 25 patients (23.1%) treated with PIP/TAZ reached a PIP serum concentration above 100 mg/L.

Clinically relevant laboratory and severity variables, including eGFR, albumin, SOFA score, and SAPS II score, were assessed at the start of antibiotic therapy and are therefore presented in Table 3 rather than in Table 1.

To interpret the significant differences in PIP levels between patients with and without organ replacement therapy, we assessed renal function as a marker of renal elimination. Median eGFR was 76 (65-107) mL/min/1.73 m^2^ in Group A and 93 (55-103) mL/min/1.73 m^2^ in Group B. eGFR was not calculated for Groups C and D, as ongoing CRRT precludes a valid interpretation of creatinine-based renal function estimates (Table 3).

Although renal elimination predominates, we also assessed bilirubin levels as a marker of biliary drug elimination. The median bilirubin serum concentrations were 0.60 (0.43–0.90) mg/dL in Group A, 0.80 (0.53–2.10) mg/dL in Group B, 0.75 (0.58–1.52) mg/dL in Group C, and 1.30 (1.10–2.10) mg/dL in Group D (Table 3).

We also compared albumin levels as a parameter for protein binding and the CLI as a marker of vascular permeability due to systemic inflammation across the subgroups. The median albumin level across all patients was 2.6 (2.3–2.9) g/dL. Only 1 out of 91 patients (1.1%) had a normal serum albumin level (defined range: 3.5–5.2 g/dL); all others had lower values.

A binary regression analysis did not allow for a significant prediction of achieving the target concentration range based on any single parameter mentioned.

## Discussion

Critically ill patients undergo several pathophysiological pharmacokinetic changes due to organ failure, intensive treatment, systemic inflammation, or pre-existing chronic diseases. Based on an altered volume of distribution on the one hand and drug elimination on the other hand, several factors must be considered when determining antibiotic therapy (Abdul-Aziz et al., 2020).

Endothelial dysfunction is a prevalent issue among critically ill patients, influencing the volume of distribution and resulting in significant changes in the serum concentration of hydrophilic antibiotics, particularly beta-lactams (Gonçalves-Per et al., 2011). In addition to a systemic inflammation caused by a cytokine storm - potentially exacerbated by secondary bacterial infections - a *SARS-CoV-2* infection can directly affect the vascular system (Xu et al., 2023). The virus infects and replicates in endothelial cells (Filbin, 2022), and alterations in various proteins involved in endothelial tight junctions have been described (Ashour, 2023). Beyond its pharmacological consequences, organ dysfunction and a worsened prognosis have been correlated with increased vascular permeability syndrome (Cordemans et al., 2012). Following the definition of the CLI, which is influenced by markers of systemic inflammation (CRP) and hypoalbuminemia (Wollborn et al., 2021), we determined CLI levels in our study population. The results indicate that permeability changes occurred in the study cohort, with a significant correlation between CLI and the severity of organ impairment, as summarized in the SOFA score. Notably, patients requiring organ replacement therapy demonstrated significantly different CLI values compared to those without organ support.

Regarding plasma protein binding, hypoalbuminemia is associated with changes in the volume of distribution and drug clearance (Abdul-Aziz et al., 2020; Roberts et al., 2013). Multiple mechanisms, including capillary leakage, high catabolism, and altered protein synthesis, may contribute to hypoalbuminemia in sepsis. While its precise impact on sepsis-related mortality remains inconsistent, hypoproteinemia - particularly in patients with *SARS-CoV-2* infection–has been associated with excessive inflammatory cytokine levels and potential hepatotoxicity (Hu et al., 2021; Ali and Kunugi, 2021; Huang et al., 2020). In line with these findings, a significant negative correlation was identified between the SOFA score and the degree of hypoalbuminemia in most evaluated patients. Some guidelines recommend albumin measurement to guide beta-lactam therapy. However, the relationship between protein levels and plasma-free drug concentration is inconsistent, as multiple competing factors, such as sedative drug interactions and increased free drug fractions leading to higher clearance, affect drug binding. This makes predicting drug levels based solely on albumin concentration unreliable (Benet and Hoener, 2002; Guilhaumou et al., 2019).

Renal elimination, which includes glomerular filtration and tubular secretion, accounts for 50%–60% of the administered PIP dose (Sörgel et al., 1994; Sinnol et al., 2018). In the context of AKI in COVID-19 patients, a recent meta-analysis reported an AKI prevalence of 46% among ICU patients, with 19% undergoing renal replacement therapy (Silver et al., 2021; Aklilu et al., 2024). Furthermore, patients on renal replacement therapy exhibit highly variable antibiotic concentrations due to multiple continuously changing factors, including therapy intensity, as well as residual endogenous renal function (Roberts et al., 2012; Abdul-Aziz et al., 2020; Roberts et al., 2021).

In the literature, a significant portion of patients receiving ECMO therapy exhibit inadequate serum PIP levels. Pharmacokinetic changes such as haemodilution and drug sequestration may contribute to these findings, alongside patient-specific factors such as multi-organ failure and disease severity (Kühn et al., 2020; Shekar et al., 2012; Donadello et al., 2015; Gomez et al., 2022).

In our retrospective analysis, we found unpredictable PIP levels, especially in patients receiving combined organ replacement therapies. While multiple variables may explain these pharmacokinetic alterations, their definitive practical impact remains unclear.

Insufficient antibiotic serum levels may negatively affect patient outcomes. Subtherapeutic serum concentrations can lead to ineffective pathogen control, while excessively high beta-lactam levels can cause adverse effects such as neurotoxicity and renal failure, necessitating precise and individualized dosing (Abdul-Aziz et al., 2020; Deshayes et al., 2017). Piperacillin is a time-dependent beta-lactam antibiotic, and the PK/PD parameter considered most relevant for efficacy is the time during which the free drug concentration exceeds the minimum inhibitory concentration (fT > MIC). However, the optimal PK/PD target for PIP in critically ill patients remains under debate. While some data suggested 100% fT > MIC for critically ill patients, others even suggested 100% fT > 4-10×MIC (Guilhaumou et al., 2019; De Waele et al., 2014). In contrast, recent evidence showed the lowest mortality in patients treated with PIP and target attainment of 100% fT > MIC without marked overexposure above 4×MIC (Richter et al., 2019; Dhaese et al., 2019; Scharf et al., 2020). However, only total piperacillin concentrations were measured in the present study; therefore, direct assessment of free-drug PK/PD target attainment was not possible. Our institutional SOP distinguishes between an empirical target range of 32–64 mg/L for other bacteria and a higher target range of 64–96 mg/L in the context of suspected or proven *Pseudomonas aeruginosa* infection. Accordingly, the broader concentration range of 32–96 mg/L should not be interpreted as a single uniform target range for all patients, but rather as the combination of two clinically distinct exposure targets. The empirical target range in our SOP is 32–64 mg/L, while the broader SOP-defined range was additionally considered to reflect higher-exposure clinical scenarios. This distinction may partly explain why patients receiving both ECMO and CRRT were particularly prone to concentrations above the desired range. The potential ramifications of this development may include the standardization of TDM in clinical practice. Nevertheless, it is important to note that TDM is still controversial and requires more research to prove its clinical usefulness, as there is no clear evidence of its beneficial effects on patients (Hagel et al., 2022).

## Conclusion

The indication, selection and dosing of antibiotics in modern intensive care medicine pose significant challenges. Multiple pathophysiological changes - affecting absorption, dilution, protein binding, and elimination - make predicting sufficient drug levels difficult. Additionally, the impact of contemporary organ replacement techniques (e.g., continuous renal replacement therapy or extracorporeal membrane oxygenation) remains highly variable. TDM is a debatable tool with potential to reduce toxicity and prevent inadequate treatment. Further evaluation of data would benefit this topic.

## Limitations

Due to the retrospective nature of this study and the limited sample size, the findings should be interpreted as observational trends rather than definitive conclusions. In addition, given the retrospective design, the absence of exact individual sampling times, and the lack of rich concentration-time data per patient, a formal concentration-time plot and a nonlinear mixed-effects population pharmacokinetic analysis were not feasible in this cohort. Antibiotic initiation was based on clinical decision-making, and while retrospective analyses provide valuable insights into clinical practice, the results cannot replace randomized controlled trials. Detailed information on concomitant medications with potential hepatic enzyme–inducing or–inhibiting effects, as well as systematic assessment of augmented renal clearance, was not available. Moreover, the daily PK fluctuations related to dynamic changes in organ function could not be evaluated. Because the first TDM sample was obtained at least 6 h after the loading dose, it should be interpreted as reflecting early exposure under near-steady-state conditions rather than fully established steady state, particularly in patients with dynamic renal dysfunction. Formal AKI classification was not feasible because urine output data required for KDIGO staging were not consistently available, and creatinine-based eGFR is not reliably interpretable during ongoing CRRT. In addition, only total piperacillin concentrations were measured, and isolate-specific MIC data were not available; therefore, target attainment could not be assessed on a pathogen-specific or free-drug basis and was instead interpreted in relation to the SOP-defined concentration targets used in routine clinical practice.

## Data Availability

The data presented in this study are available on reasonable request from the corresponding author. The data are not publicly available due to data protection regulations (GDPR).
